# A Comprehensive MicroRNA Expression Profile Related to Hypoxia Adaptation in the Tibetan Pig

**DOI:** 10.1371/journal.pone.0143260

**Published:** 2015-11-16

**Authors:** Bo Zhang, Yangzong Qiangba, Peng Shang, Zhixiu Wang, Jun Ma, Liyuan Wang, Hao Zhang

**Affiliations:** 1 National Engineering Laboratory for Animal Breeding, China Agricultural University, Beijing, People's Republic of China; 2 College of Agriculture and Animal Husbandry, Tibet University, Linzhi, People's Republic of China; Kunming University of Science and Technology, CHINA

## Abstract

Tibetan pigs live between 2500 and 4300 m above sea level on the Tibetan Plateau, and are better adapted to hypoxia than lowland pigs. MicroRNAs (miRNAs) are involved in a wide variety of cellular processes; however, their regulatory role in hypoxia adaptation remains unclear. In this study, miRNA-seq was used to identify differentially expressed miRNAs (DE miRNAs) in the cardiac muscle of Tibetan and Yorkshire pigs, which were both raised in high elevation environments. We obtained 108 M clean reads and 372 unique miRNAs, which included 210 known porcine miRNAs, 136 conserved in other mammals, and 26 novel pre-miRNAs. In addition, 20 DE miRNAs, including 10 up-regulated and 10 down-regulated miRNAs, were also found after comparison between Tibetan and Yorkshire pigs. We predicted miRNA targets based on differential expression and abundance in the two populations. Furthermore, the results of a Kyoto Encyclopedia of Genes and Genomes pathway analysis suggested that DE miRNAs in Tibetan and Yorkshire pigs are involved in hypoxia-related signaling pathways such as the mitogen-activated protein kinase, which is the mechanistic target of rapamycin, and the vascular endothelial growth factor, as well as cancer-related signaling pathways. Five DE miRNAs were randomly selected to validate the results of miRNA-seq using real-time polymerase chain reaction, and the results corresponded to those from the miRNA-seq, confirming that deep-sequencing methods are feasible and efficient. In our study, we identified various previously unknown hypoxia-related miRNAs in pigs, and the data obtained suggest that hypoxia-related miRNA expression patterns are significantly altered in the Tibetan pig compared to other species. Therefore, DE miRNAs may play an important role in organisms that have adapted to hypoxic environments.

## Introduction

MicroRNAs (miRNAs) are small non-coding RNA molecules found in plants, animals, and viruses, and are widely believed to repress gene expression by binding to specific mRNA sequences [1–3]. Furthermore, miRNAs can be transferred to the nucleus [4] and guide the remodeling of chromatin and silencing of gene transcription [5], resulting in *de novo* DNA methylation [6]. Since miRNAs play different roles in multiple aspects of cellular function, it is not surprising that they are involved in hypoxia-related gene regulation. Kulshreshtha et al. [7] reported that experiencing hypoxia changes miRNA profiles in various cell types, and is affects the hypoxia-inducible factor (HIF) signal pathway. Moreover, several hypoxia-regulated miRNAs play roles in cell survival in hypoxic environments and have been implicated in the regulation of both upstream and downstream HIF signaling pathways, e.g., miR-20b and miR-17-92 clusters, while miR-199a regulates *HIF-1α* under hypoxic conditions [8–10], and miR-107, miR-210, miR-373, miR-23, miR-24, and miR-26 are induced by HIFs [7, 11, 12].

The Tibetan pig inhabits high-altitude regions (2500–4300 m) on the Qinghai-Tibet Plateau in southwestern China, and is well adapted to extreme elevations [13–15]. It is an ideal animal model for investigating the molecular mechanisms of hypoxia adaptation. Hypoxia could causes myocardial hypolasia, cardiomyopathy and reduced heart rate [16, 17]; therefore, identifying the regulatory mechanism of miRNAs in Tibetan pig cardiac muscle would elucidate the animals’ responses and molecular adaptation to hypoxic conditions, as well as enable us to determine not only the genes involved but also understand the regulation of specific hypoxia-related miRNAs. In this study, we conducted a comprehensive miRNA expression profile using miRNA-seq in the cardiac muscle of Tibetan and Yorkshire pigs raised in upland environments, established an overview of differential miRNA expression, and identified key miRNAs involved in hypoxia adaptation.

## Materials and Methods

### Sample preparation and RNA isolation

Sixteen 6-month-old castrated boars from populations of Tibetan pigs (TPs, n = 8) and Yorkshire pigs (YPs, n = 8) that were raised at the experimental farm of the Tibet Agriculture and Animal Husbandry College (Linzhi, 3000 m above sea level) were slaughtered and sampled. Cardiac tissue samples were immediately frozen in liquid nitrogen and stored at -80°C until RNA extraction. Total RNA for miRNA sequencing was extracted using TRIzol reagent (Invitrogen, San Diego, CA, USA) according to the manufacturer’s protocol. Extract quality was checked using a NanoDrop^™^ Biophotometer 2000 (Thermo Fisher Scientific Inc., West Palm Beach, FL, USA); a 260/280 nm absorbance ratio of 1.8–2.0 indicated a pure RNA sample. Equal quantities of the RNA extracted from the cardiac tissue of four pigs in each population were pooled into one. Thus, we had two duplicate samples in Tibetan pigs, two duplicate samples in Yorkshire pigs. Specific details are provided in S1 Table. The experiments were approved by the animal welfare committee of the State Key Laboratory for Agro-Biotechnology of the China Agricultural University (Approval number XK257). Pig farming at Linzhi was permitted, and the field study did not involve endangered or protected species.

### Library construction and sequencing

Three micrograms of total RNA per sample was used for the construction of a small RNA library. Sequencing libraries were generated using NEBNext^®^ Multiplex Small RNA Library Prep Set for Illumina^®^ (NEB Ltd., UK) following the manufacturer’s recommendations, and index codes were added to attribute sequences to each sample. Polymerase chain reaction (PCR) products were purified on an 8% polyacrylamide gel (100 V, 80 min). DNA fragments corresponding to 140–160 bp (the length of small noncoding RNA plus 3′ and 5′ adaptors) were recovered and dissolved in 8 μL of elution buffer. The quality of the library was assessed using an Agilent Bioanalyzer 2100 system with DNA high-sensitivity chips. Clustering of the index-coded samples was performed on a cBot Cluster Generation System using a TruSeq SR Cluster Kit v3-cBot-HS (Illumina Inc.), according to the manufacturer’s protocol. After cluster generation, the library preparations were sequenced on a HiSeq^™^ 2000 platform and 50-bp paired-end reads were generated. The microRNA sequencing profile data were deposited in the Gene Expression Omnibus with the accession number GSE71550.

### Data analysis

Clean reads were obtained from raw data after strictly eliminating low-quality reads, trimming adaptor sequences, and mapping (allowing two end-nucleotide mismatches) to reference pig genomic sequences (S scrofa10.2.72). The matched reads ranging from 15 to 35 bp were used as a query against non-coding RNA data (rRNA, snoRNA, tRNA, etc.). After excluding reads matching non-miRNA databases, the remaining sequences were regarded as potential miRNA reads. Precursors of candidate miRNAs that had been assembled from the short-read library were then searched against the pig genome to determine their location on the chromosome. Known pig miRNAs were identified by mapping the matched mature miRNAs in miRBase20.0 to pig genome sequences. All of the unannotated reads that matched the pig genomic sequences were analyzed using miRDeep2 to predict novel miRNAs. In addition, all candidate novel miRNAs were classified as conserved or pig-specific, according to their sequence conservation among the candidate species.

The microRNA read counts were normalized by the trimmed mean of TPM (transcript per million) normalization method in the edgeR package [18, 19]. *P*-values to compare miRNA expression levels were calculated using DEGseq software. Fold change = log_2_ (normalized read counts in TP/normalized read counts in YP).

### Bioinformatic analyses

For the primary analysis, the retained reads (clean reads) were mapped to the pig genome using the custom mapping tools including the Bowtie 0.12.5 package [20] and miRDeep2 (version mirdeep2_0_0_2) [21]. The pig genome sequence was obtained from ftp://ftp.ensembl.org/pub/release-72/fasta/sus_scrofa/dna/Sus_scrofa.Sscrofa10.2.72.dna.toplevel.fa.gz. Clean reads were initially mapped to miRBase20.0 (www.mirbase.org/) [22] to identify known miRNAs and miRNA hairpins previously characterized [23–25]. Unmappable reads were annotated and classified by referencing noncoding RNAs in the NONCODE (version3.0) [26] and Rfam 11.0 databases. Since there is no complete pig miRNA dataset in miRBase, we obtained known mature miRNA for pigs, humans, mice, cows, and sheep (326, 257, 1908, 783, and 153, respectively) from miRBase 20.0 for use in miRDeep (mirdeep2_0_0_2) [21] to predict novel pig miRNA. Venn diagrams were prepared using the Venn diagram function in R, based on lists of novel and known miRNAs identified in each group. DEGseq was used to identify DE miRNAs in TPs and YPs. A volcano plot was generated based on log_2_ normalized read counts and -log10 (*P*-value). The August 2010 release of the microRNA target detection software miRanda [27] was used for target gene prediction. Kyoto Encyclopedia of Genes and Genomes (KEGG) pathway [28] annotations of the miRNA targets were found using the DAVID (Database for Annotation, Visualization, and Integrated Discovery) gene annotation tool[29].

### Stem-loop reverse-transcription quantitative PCR (RT-qPCR) to validate miRNA expression

A stem-loop RT-qPCR assay was conducted to measure the quantity of specific mature miRNA expression and validate the DE miRNAs [30]. Briefly, a miRcute miRNA First-Strand cDNA Synthesis Kit (KR201, Tiangen Biotech Co. Ltd., Beijing, China) was used for reverse transcription and real-time PCR according to the manufacturer’s instructions. Pig 5S and U6 snRNA was used as an internal control. All of the reactions were run in triplicate, and a mixture of every cDNA sample was used for calibration. Primer sequences for miRNA amplification are listed in S2 Table. A BioRad CFX96 (Bio-Rad, CA, USA) was used to perform the RT-qPCR with a SYBR^®^ Green PCR Master Mix (FP401, Tiangen Biotech Co. Ltd.). Eight biological samples consisted of 8 single individuals for each group in the measurement. Relative miRNA expression levels were calculated using the 2^-ΔΔCT^ method [31].

## Results

### Overview of miRNA transcriptome profiles in pig cardiac muscle

We obtained 21.2 M to 32.0 M clean reads from each of the four small RNA libraries (S3 Table). The number of reads with 20–24 nt was greater than that of those with shorter or longer sequences (S1 Fig). Approximately half of the sequences were 22 nt in length, which coincided with the known specifity of Dicer processing and characteristics of mature miRNAs [32, 33]. Approximately 87% of the clean reads could be mapped to the pig genome (Sscrofa10.2.72, S3 Table).

### Identification of pig miRNAs

**Fig 1:**
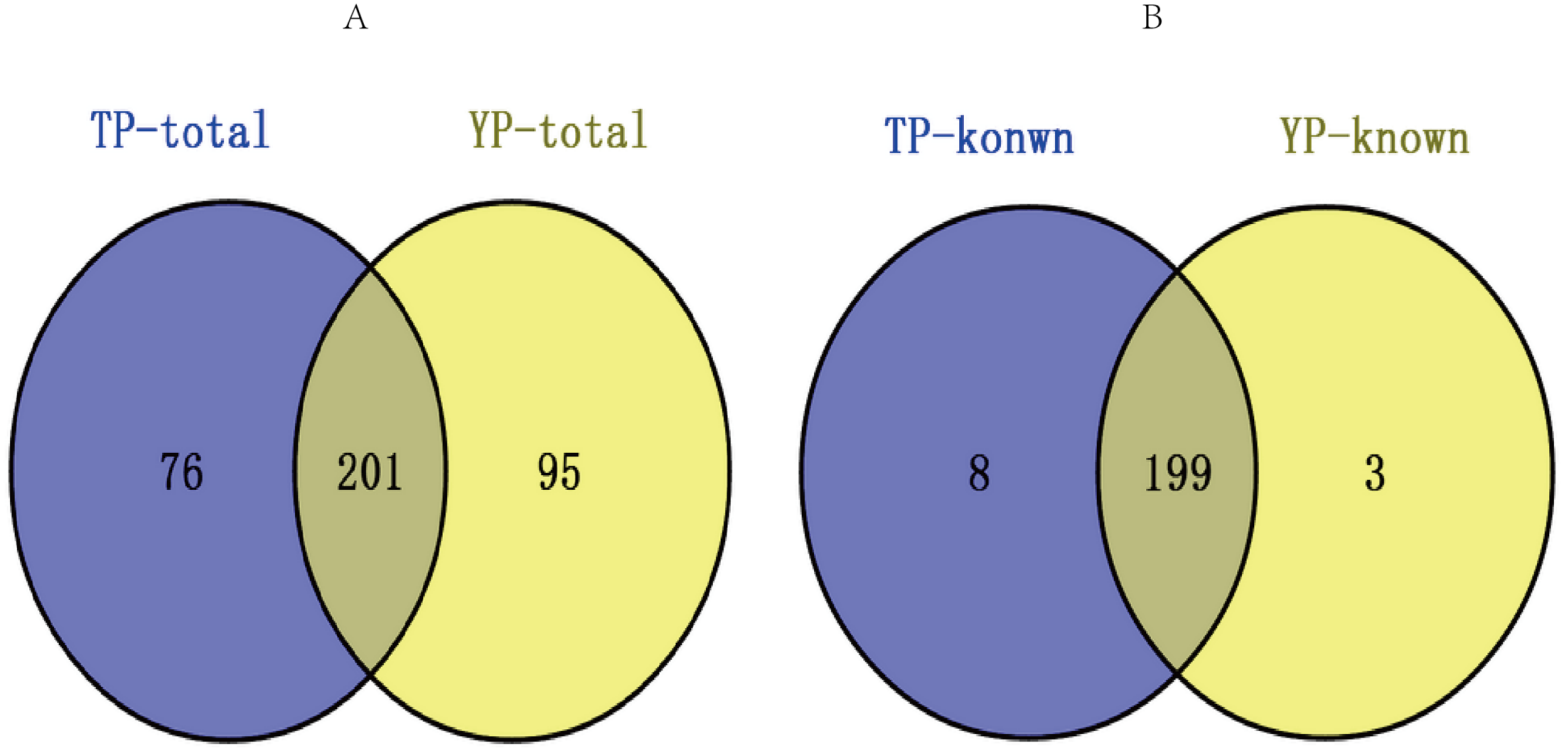
Venn diagrams demonstrating relationships among miRNA in Tibetan and Yorkshire pigs. (A) Venn diagram for total miRNAs (contained novel miRNAs and known miRNAs). (B) Venn diagram for known porcine miRNAs. TP and YP miRNAs marked in blue and yellow cycle, respectively. Tibetan pigs (TP) and Yorkshire pigs (YP).

The annotated sequences were analyzed according to the data from miRBase20.0 (containing 326 mature miRNAs and 280 hairpin precursors). According to the results, 192–202 mature miRNAs and 230–234 hairpin precursors were identified (S3 Table). The remaining annotated sequences were compared using the Rfam and ncRNA databases after removal of the cellular structural RNAs, such as rRNAs, snoRNAs, snRNAs, scRNAs, and tRNAs. This revealed that nearly all remaining sequences were known miRNAs (S2 Fig). After filtering these data, 372 unique miRNAs, comprising 210 known porcine miRNAs (S4 Table) and 162 predicted candidate pre-miRNAs (S5 Table), were identified. According to this program for miRDeep2 software we identified the miRNAs that satisfy the requirements to be classified as novel, and the secondary structure of potential precursor and the mature miRNA, star sequences and loops were reliable. The TP and YP both expressed 201 miRNAs and in which 199 were known porcine miRNAs (Fig 1). In the 162 candidate miRNAs, 136 were conserved in other mammals (human, mouse, cow, and sheep) while the other 26 were considered as novel pre-miRNAs (S5 Table). The chromosomal locations of known and novel pre-miRNAs were determined using the pig reference genomic sequence. All miRNAs were aligned against autosomes or the X chromosome (S4 and S5 Tables).

### DE miRNAs in TPs and YPs

**Fig 2:**
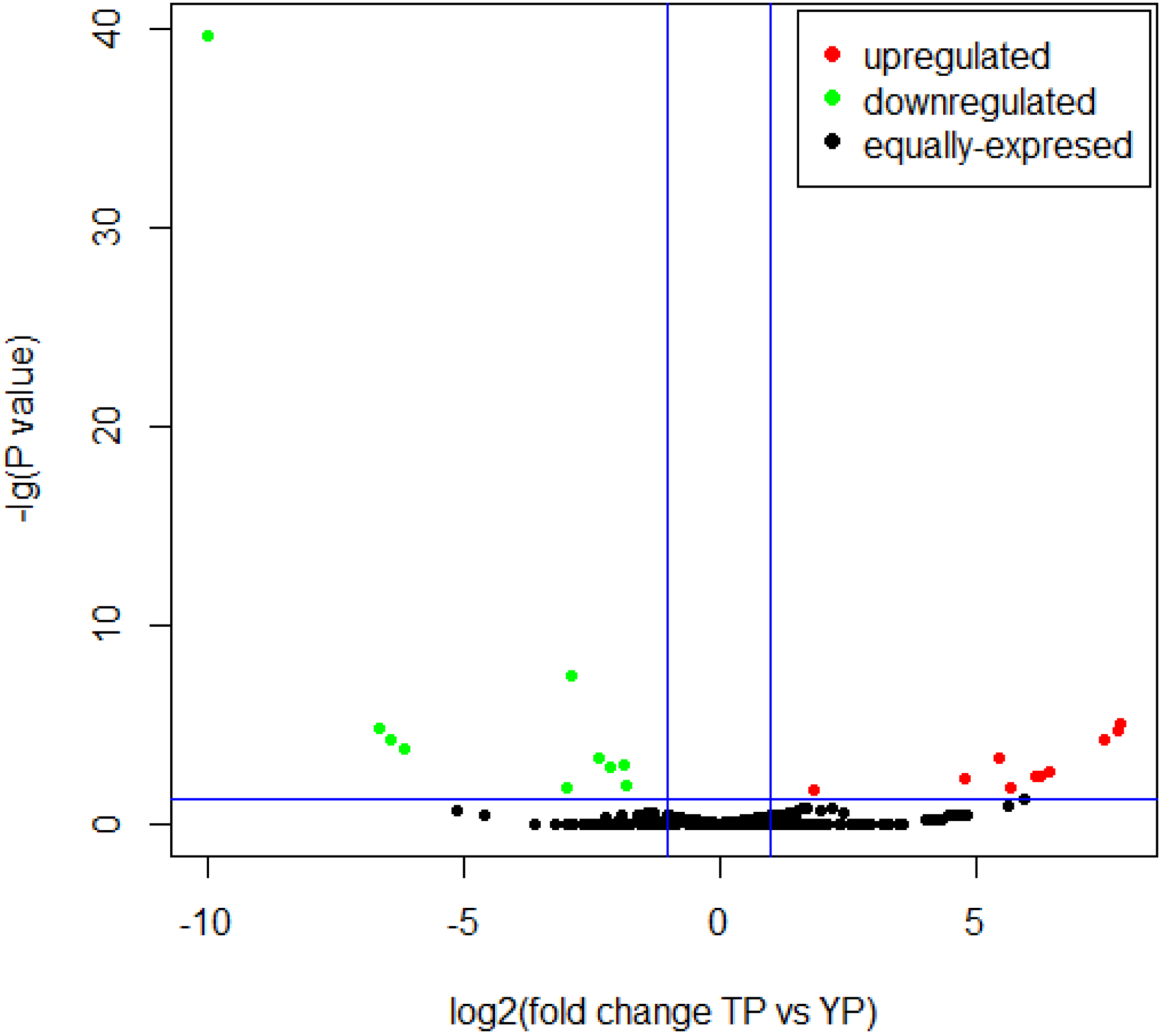
Volcano plot displaying differentially expressed miRNAs identified using miRNA-seq in Tibetan and Yorkshire pigs. The y-axis represents the mean expression value of log10 (*P*-value) and the x-axis displays the log_2_-fold change value. Up-regulated and downregulated miRNAs are shown in red and green, respectively. Black dots indicate genes with no significant change in expression.

Based on the criteria of a fold change either ≥2 or ≤0.5 and *P* ≤ 0.05, 20 DE miRNAs were identified when comparing 372 miRNAs between TP and YP (Fig 2 and S6 Table). Of these, 10 miRNAs (ssc-miR-210, ssc-miR-1343, 12_3058, ssc-miR-676-5p, GL894044.2_23796, 1_4279, 13_5125, ssc-miR-194b-5p, ssc-miR-142-5p, and ssc-miR-421-5p) were up-regulated and 10 (ssc-miR-101, 1_1126, 4_13655, GL892805.1_27591, ssc-miR-320, ssc-miR-7136-5p, ssc-miR-214, ssc-miR-10b, 7_17790, and ssc-miR-206) were downregulated in the TP relative to the YP.

### Functional analysis of DE miRNAs

Using miRanda software and the Ensemble database, 2389 and 2295 target genes were predicted from the 10 up-regulated and 10 downregulated miRNAs, respectively (S7A and S7B Table). The predicted target genes were classified in order to identify pathways that were actively regulated by miRNAs, according to the KEGG functional annotations made using DAVID (S8 Table, Tables 1 and 2). Thirteen putative target genes of up-regulated miRNAs enriched the mammalian target of the rapamycin (mTOR) signaling pathway (S3 Fig), which can be induced to enhance angiogenesis in response to hypoxia [34]. The mitogen-activated protein kinase (MAPK) signaling pathway was enriched according to 47 putative target genes of the up-regulated miRNAs and 53 putative target genes of the downregulated miRNAs (S4 Fig). The MAPK signaling pathway participates in the activation of *HIF*s and is involved in various cellular functions, including cell proliferation, differentiation, and migration [35].

**Table 1:** The Kyoto Encyclopedia of Genes and Genomes (KEGG) pathways enriched for targets of the 10 up-regulated miRNAs in Tibetan pigs.

Signaling pathway term	Count	*P*-value	Benjamini
Phosphatidylinositol signaling system	22	7.16E-04	0.128
Endocytosis	37	1.66E-02	0.799
Chondroitin sulfate biosynthesis	8	2.40E-02	0.787
Inositol phosphate metabolism	14	2.87E-02	0.751
O-Glycan biosynthesis	9	4.54E-02	0.831
mTOR signaling pathway	13	4.67E-02	0.782
Ubiquitin mediated proteolysis	27	5.27E-02	0.772
MAPK signaling pathway	47	5.95E-02	0.769
Pancreatic cancer	16	6.32E-02	0.750
RNA polymerase	8	8.04E-02	0.798
Apoptosis	18	8.31E-02	0.778
B cell receptor signaling pathway	16	8.47E-02	0.756
Cell cycle	24	8.67E-02	0.736
N-Glycan biosynthesis	11	9.24E-02	0.734
Renal cell carcinoma	15	9.36E-02	0.714

**Table 2:** Kyoto Encyclopedia of Genes and Genomes (KEGG) pathways enriched for targets of the 10 downregulated miRNAs in Tibetan pigs.

Signaling pathway term	Count	*P*-value	Benjamini
Long-term depression	23	6.11E-05	0.011
Endometrial cancer	16	2.76E-03	0.229
Pathways in cancer	63	2.86E-03	0.164
MAPK signaling pathway	53	3.27E-03	0.143
Vascular smooth muscle contraction	27	3.32E-03	0.117
Focal adhesion	42	3.71E-03	0.110
Non-small cell lung cancer	16	4.12E-03	0.105
ErbB signaling pathway	22	4.91E-03	0.109
Phosphatidylinositol signaling system	19	8.15E-03	0.157
*VEGF* signaling pathway	19	9.42E-03	0.163
Chronic myeloid leukemia	19	9.42E-03	0.163
B cell receptor signaling pathway	19	9.42E-03	0.163
GnRH signaling pathway	23	9.96E-03	0.157
Renal cell carcinoma	18	1.02E-02	0.148
Inositol phosphate metabolism	15	1.05E-02	0.142
Melanoma	18	1.17E-02	0.146
Insulin signaling pathway	29	1.21E-02	0.141
Pancreatic cancer	18	1.35E-02	0.147
Toll-like receptor signaling pathway	23	1.42E-02	0.146
Protein export	5	1.47E-02	0.144
Glioma	16	1.82E-02	0.166
Endocytosis	36	2.04E-02	0.176
Apoptosis	20	2.11E-02	0.174
Melanogenesis	22	2.17E-02	0.171
Chemokine signaling pathway	36	2.56E-02	0.191
Gap junction	20	2.64E-02	0.189
Fc epsilon RI signaling pathway	18	2.87E-02	0.197
Colorectal cancer	19	2.92E-02	0.193
Wnt signaling pathway	30	2.92E-02	0.187
Fc gamma R-mediated phagocytosis	20	4.84E-02	0.283
Prostate cancer	19	4.88E-02	0.277
Adherens junction	17	4.92E-02	0.271
T cell receptor signaling pathway	22	5.13E-02	0.274
Small cell lung cancer	18	5.42E-02	0.279
Basal cell carcinoma	13	5.98E-02	0.296
Progesterone-mediated oocyte maturation	18	6.54E-02	0.312
*Vibrio cholerae* infection	13	6.72E-02	0.312
Epithelial cell signaling in *Helicobacter pylori* infection	15	6.74E-02	0.305
Sphingolipid metabolism	10	7.13E-02	0.313
Notch signaling pathway	11	9.34E-02	0.384
Axon guidance	24	9.60E-02	0.385

Interestingly, the renal cell carcinoma pathway was enriched by the identification of 15 putative target genes of the downregulated miRNAs (S8 Table and S5 Fig). In this case *EPAS1* (Endothelial Per-Arnt-Sim (PAS) domain protein 1), *EGLN3* (EGL-nine homolog-3), *VEGFC* (vascular endothelial growth factor C), and *EGLN1* (EGL-nine homolog-1) have been reported to have strong high-altitude selective signatures in the TP [36, 37]. It was noteworthy that 63 target genes of the downregulated miRNAs in the TP belonged to cancer-related pathways (S6 Fig). These pathways are involved in sustained angiogenesis, proliferation, genomic damage, and inhibition of differentiation, all of which are closely related to cancer growth and development under hypoxic conditions [38].

### Validation of DE miRNAs

**Fig 3:**
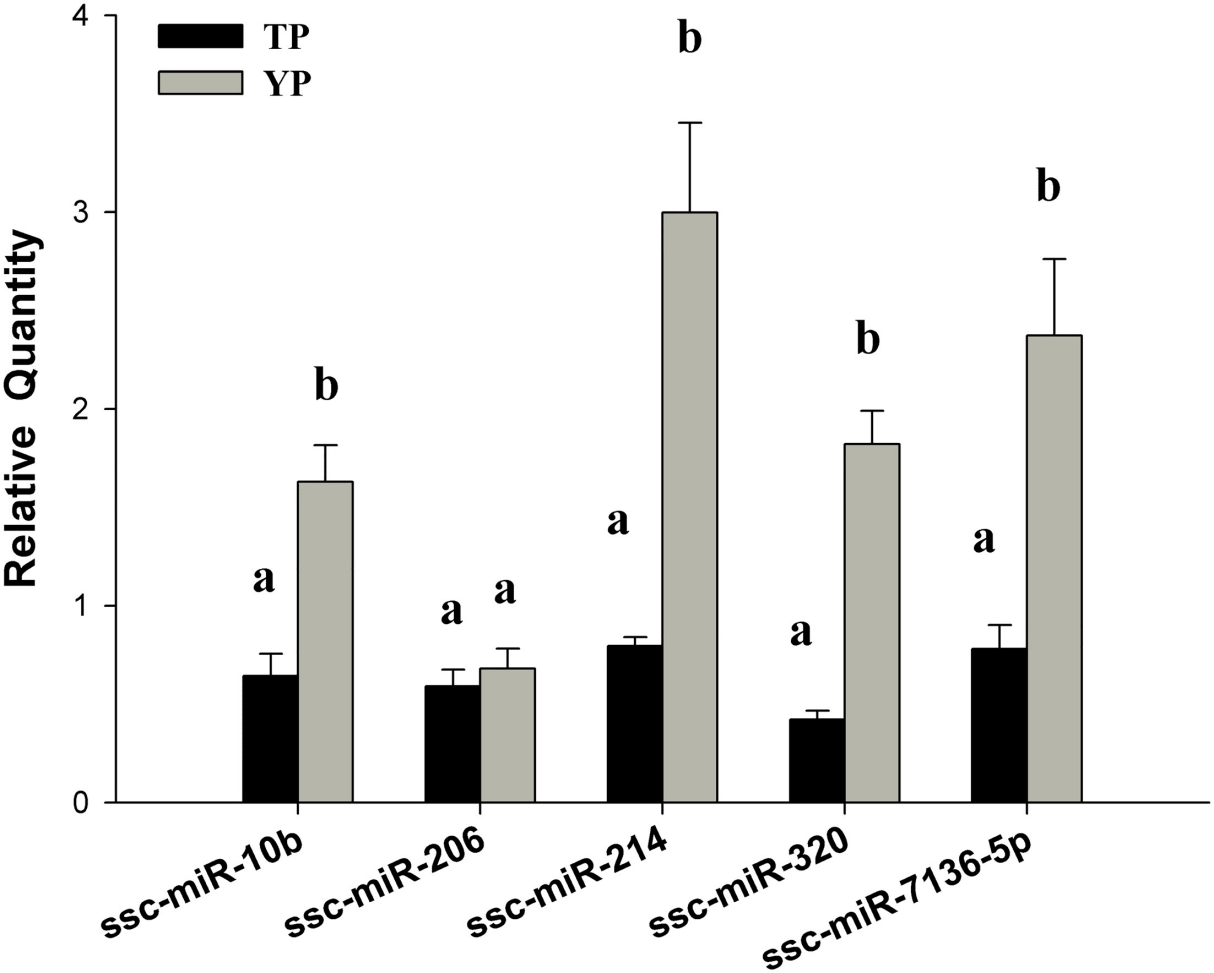
Five cardiac tissue differentially expressed (DE) miRNAs validated by reverse-transcription quantitative polymerase chain reaction. Relative expression levels of DE miRNAs. Upper letters (a, b) on bars denote significantly different expression levels in the same miRNA (*P* < 0.05).

Expression levels of five miRNAs (ssc-miR-10b, ssc-miR-206, ssc-miR-214, ssc-miR-320, and ssc-miR-7136-5p) in the cardiac tissues of TPs and YPs were assessed by stem-loop qPCR. The results showed that four of the five miRNAs had significantly higher expression levels in TPs than in YPs (Fig 3), which was consistent with the miRNA-seq data. The different expression trends observed in the miRNA-seq data and from the RT-qPCR were uniform for five miRNAs in the TPs and YPs (S9 Table).

## Discussion

High-altitude populations of pigs have evolved genetic adaptations that allow for survival in extremely hypoxic environments. Previous reports have demonstrated that TPs in particular exhibit a distinct suite of phenotypic and physiological traits, including thin-walled pulmonary vascular structures and high blood flow [13, 14, 39], which are shaped by natural and artificial selection, allowing them to adapt to high-altitude environments [40]. The relatively recently identified miRNAs constitute a novel class of master regulators that control gene expression, and are responsible for a variety of normal and pathological cellular processes. High-throughput sequencing has generated new insights into global gene expression, and provided evidence for the complexity of the mammalian transcriptome and allowed for the development of miRNAomics [41].

In this study, miRNA profiles in the cardiac tissues of TPs and YPs, both adapted to a high-altitude environment, were obtained using next-generation sequencing. We identified 20 DE miRNAs in the two pig breeds, of which ssc-miR-214 downregulated expression in the TP. Previous studies have demonstrated that alcohol depresses glutathione reductase (*GSR*) and P450 oxidoreductase (*POR*) gene expression by the up-regulation of miR-214 that induces oxidative stress, which plays an important role in responses to hypoxia [42, 43].

The most notably up-regulated miRNA in the TP (FC = 230), ssc-miR-210, is induced by HIF1α under hypoxic conditions in mice [11, 12]. miR-210 is located on an intron of a noncoding RNA, transcribed from AK123483 on the human chromosome 11p15.5, and its expression correlates with *VEGF* regulation and angiogenesis in breast cancer patients [44]. Using bioinformatics analyses, miR-210-regulated factors have been found to be implicated in DNA repair pathways [11], and has been found to play roles in modulating the expression of proteins involved in the homology-dependent repair and nucleotide-excision repair pathways, and reverses cellular DNA damage during hypoxia. Our results demonstrate that miR-210 is expressed more in the TP than in the YP, which suggests that miR-210 could modulate key factors that are related to cellular or organic hypoxia adaptation pathways in the TP.

Based on these functional pathways, 100 putative target genes (including 47 up-regulated miRNAs and 53 downregulated miRNAs) were enriched in the MAPK signaling pathway (S4 Fig). The MAPK pathway may up-regulate *HIF* activity, and plays an essential role in tumor growth and transformation that depends on angiogenesis and changes in glucose metabolism [35]. The expression of a gene that is involved in cancer and MAPK signaling pathways, *FOS* (FBJ murine osteosarcoma viral oncogene homolog) (S7B Table), is increased by miR-101 under hypoxic conditions [45, 46]. Furthermore, *FOS* may be a potential target of ssc-miR-101, which was downregulated in the TP in this study.

**Fig 4:**
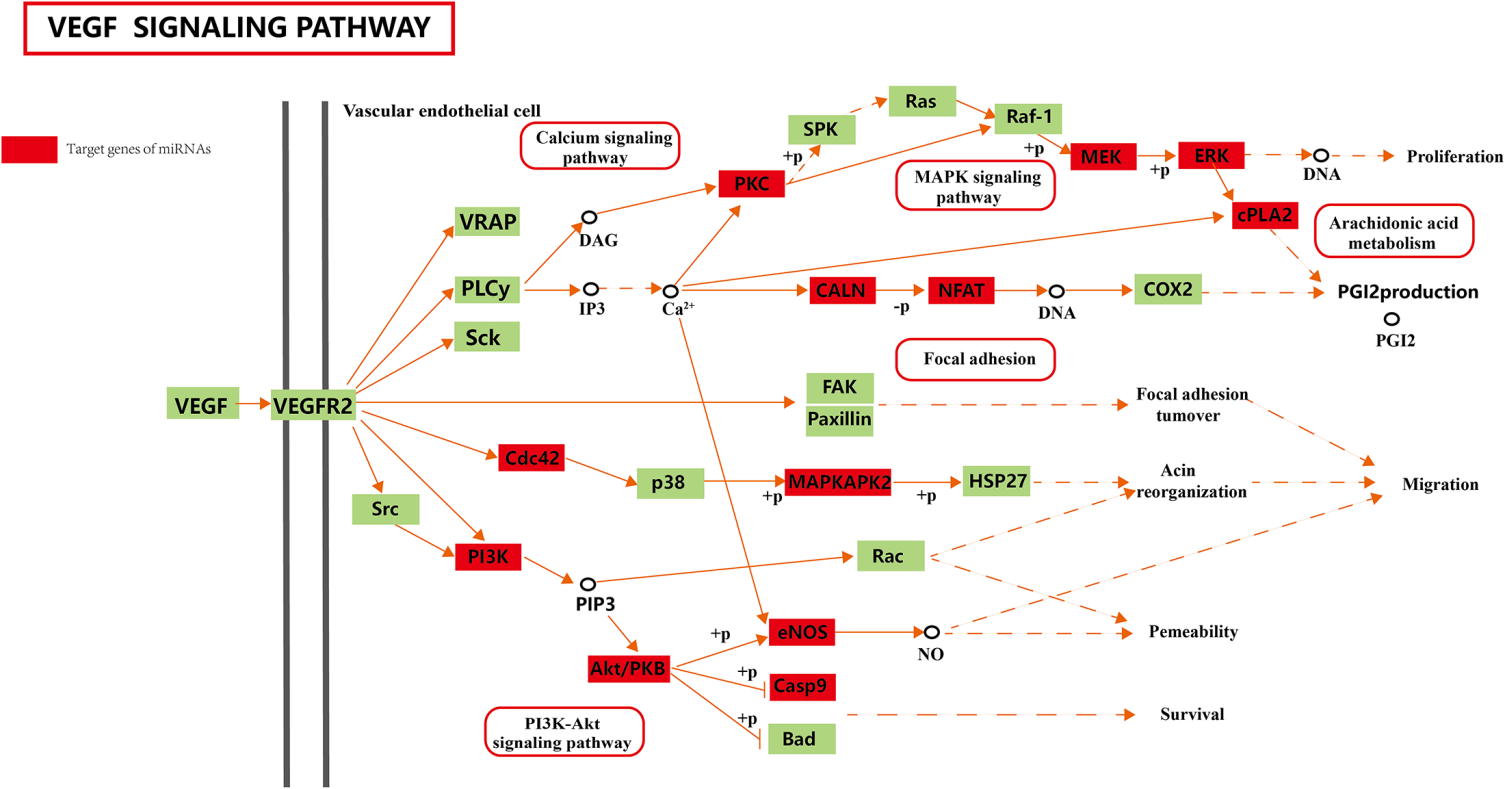
The *VEGF* signaling pathway enriched by 19 putative target genes of downregulated miRNAs. Red boxes represent the target genes of miRNAs.

The *VEGF* signaling pathway regulates angiogenesis, endothelial cell growth, proliferation, and migration, affects the permeability of blood vessels [47, 48], and plays important roles in hypoxia adaptation. In our study, 19 putative target genes that are regulated by downregulated miRNAs (ssc-miR-101, ssc-miR-7136-5p, ssc-miR-214, ssc-miR-10b, ssc-miR-206, and ssc-miR-320) were found to be involved in the *VEGF* signaling pathway (Fig 4). *VEGF* up-regulation by HIF is accompanied by the stability of its mRNA and an increase in translation, which are essential for hypoxia-related angiogenesis. Another important pathway that was targeted by downregulated miRNAs in cardiac tissue is involved in vascular smooth muscle contraction. This pathway is associated with energy metabolism, including soluble guanylyl cyclase [49] and potassium and calcium channels (such as sarcoplasmic reticulum calcium ATPase) [50, 51].

Four DE miRNAs (ssc-miR-210, ssc-miR-1343, ssc-miR-142-5p, and ssc-miR-421-5p) that are involved in the renal cell carcinoma pathway were upregulated in TPs, and the corresponding target genes were *EPAS1*, *EGLN3*, *EGLN1*, and *VEGFC*. Both *EPAS1* and *EGLN1* are closely associated with the high-altitude adaptation of Tibetan populations [37], and are involved in hypoxia pathways as key regulators during chronic hypoxia [52]. Genetic variation in *EPAS1* and *EGLN1* is associated with Hb levels and high-altitude adaptation in Tibetan populations [36, 53, 54]. MiRNA-pathway-enrichment analysis revealed that the miRNAs might contribute to high-altitude adaptation by participating in signaling pathway in TPs. *VEGFC* is involved in two signaling pathways: renal cell carcinoma and mTOR. Hypoxia signaling (in particular *HIF-1α)* regulates the expression of *VEGFC*, which is one of the key lymphangiogenic factors [55]. Liang et al. [56] reported an associative correlation between *HIF-1α* and *VEGFC* in cancer. Here, four DE miRNAs related to hypoxia were found to have high expression levels in TPs, suggesting that miRNAs and target-gene regulation enabled the TP to adapt to a hypoxic environment.

In conclusion, this study significantly increased the number of hypoxia-related miRNAs known in the pig, and identified miRNAs with significantly altered in the TP. Using miRNA-seq, 372 miRNAs were found in the cardiac tissues of pigs living at high altitudes. Twenty DE miRNAs were identified and subjected to bioinformatics functional analyses. The results suggest that 20 miRNAs involved in the mTOR signaling pathway, the MAPK signaling pathway, the renal cell carcinoma pathway, various cancer pathways, the *VEGF* signaling pathway, and the vascular smooth muscle contraction pathway play regulatory roles in hypoxia adaptation in the TP. This study provides new insights that will advance the study adaptation to hypoxia in humans and other mammals living at high altitude. The next step, the functional mechanism of the DE miRNA regulating the hypoxia adaptation in the Tibetan pig was to be investigated.

## Supporting Information

S1 Fig Distribution of different RNA classes. (PDF)

S2 Fig Venn diagrams demonstrating relationships between miRNAs in Tibetan and Yorkshire pigs. (PDF)

S3 Fig Mammalian target of the rapamycin (mTOR) signaling pathway enriched by 13 putative target genes of upregulated miRNAs. (PDF)

S4 Fig Mitogen-activated protein kinase (MAPK) signaling pathway enriched by 47 putative target genes of upregulated miRNAs (A) and 53 putative target genes of downregulated miRNAs (B). (PDF)

S5 Fig Renal cell carcinoma pathway enriched by 15 putative target genes of upregulated miRNAs. (PDF)

S6 Fig Cancer pathways enriched by 63 putative target genes of downregulated miRNAs. (PDF)

S1 Table Datasets used in the study. (XLSX)

S2 Table Primers for five miRNAs in reverse-transcription quantitative polymerase chain reactions. (XLSX)

S3 Table miRNA sequencing results. (XLSX)

S4 Table Characteristics of 210 known porcine miRNAs. (XLSX)

S5 Table Characteristics of 136 conserved miRNAs in other mammals and 26 novel pre-miRNAs. (XLSX)

S6 Table Differentially expressed cardiac tissue miRNAs in Tibetan and Yorkshire pigs. (XLSX)

S7 Table Predicting target genes of differentially expressed cardiac tissue miRNAs. (XLSX)

S8 Table Kyoto Encyclopedia of Genes and Genomes (KEGG) pathways enriched for targets of differentially expressed cardiac tissue miRNAs in Tibetan and Yorkshire pigs. (XLSX)

S9 Table Log of the expression ratios obtained from reverse-transcription quantitative polymerase chain reactions and miRNA-seq data. (XLSX)
