# Supplementary figures and images for: A Comprehensive MicroRNA Expression Profile Related to Hypoxia Adaptation in the Tibetan Pig
Source: PLoS One. 2015 Nov 16;10(11):e0143260. doi: 10.1371/journal.pone.0143260 (PMC4646468; doi:10.1371/journal.pone.0143260)

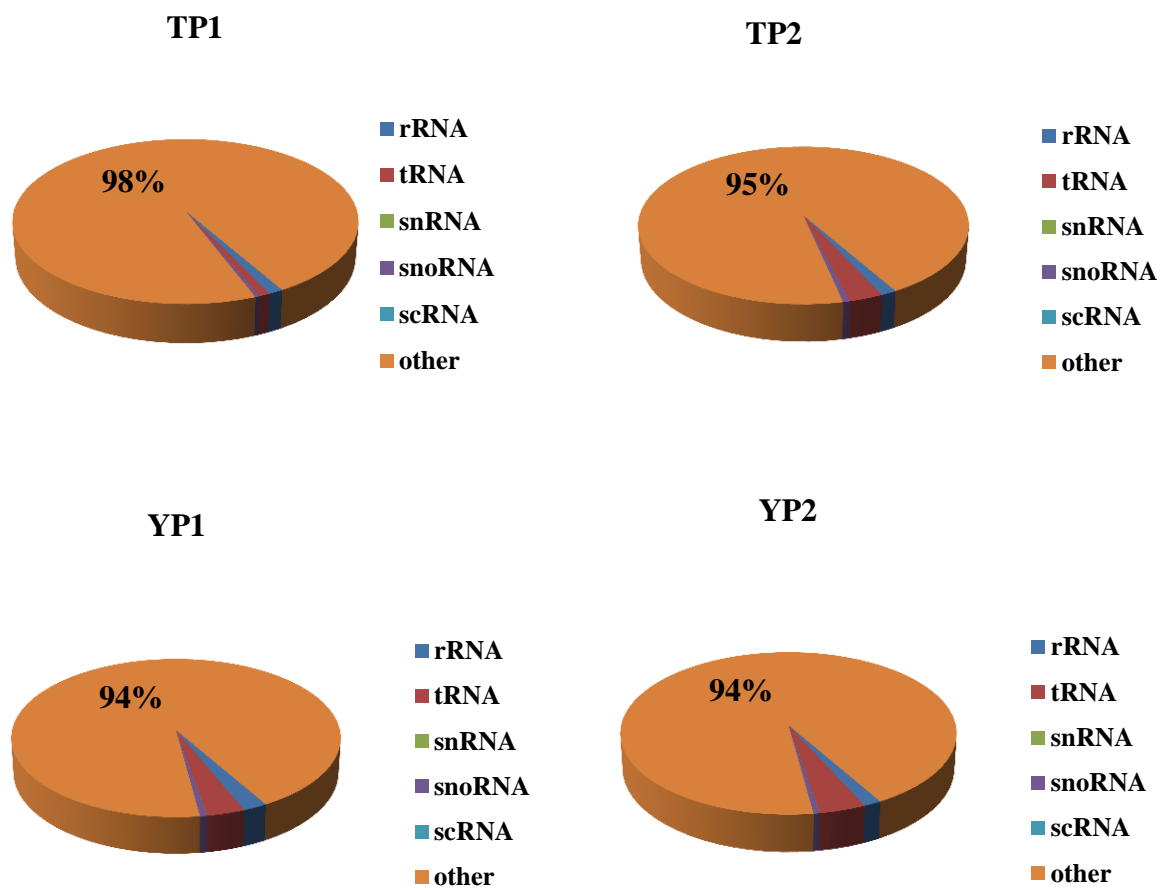

**S1 Fig. Distribution of different RNA classes.**

Supplement: S1 Fig — (PDF) [file pone.0143260.s001.pdf]
