# Supplementary material for: A Comprehensive MicroRNA Expression Profile Related to Hypoxia Adaptation in the Tibetan Pig
Source: PLoS One. 2015 Nov 16;10(11):e0143260. doi: 10.1371/journal.pone.0143260 (PMC4646468; doi:10.1371/journal.pone.0143260)
Supplement: S2 Fig — (PDF) [file pone.0143260.s002.pdf]

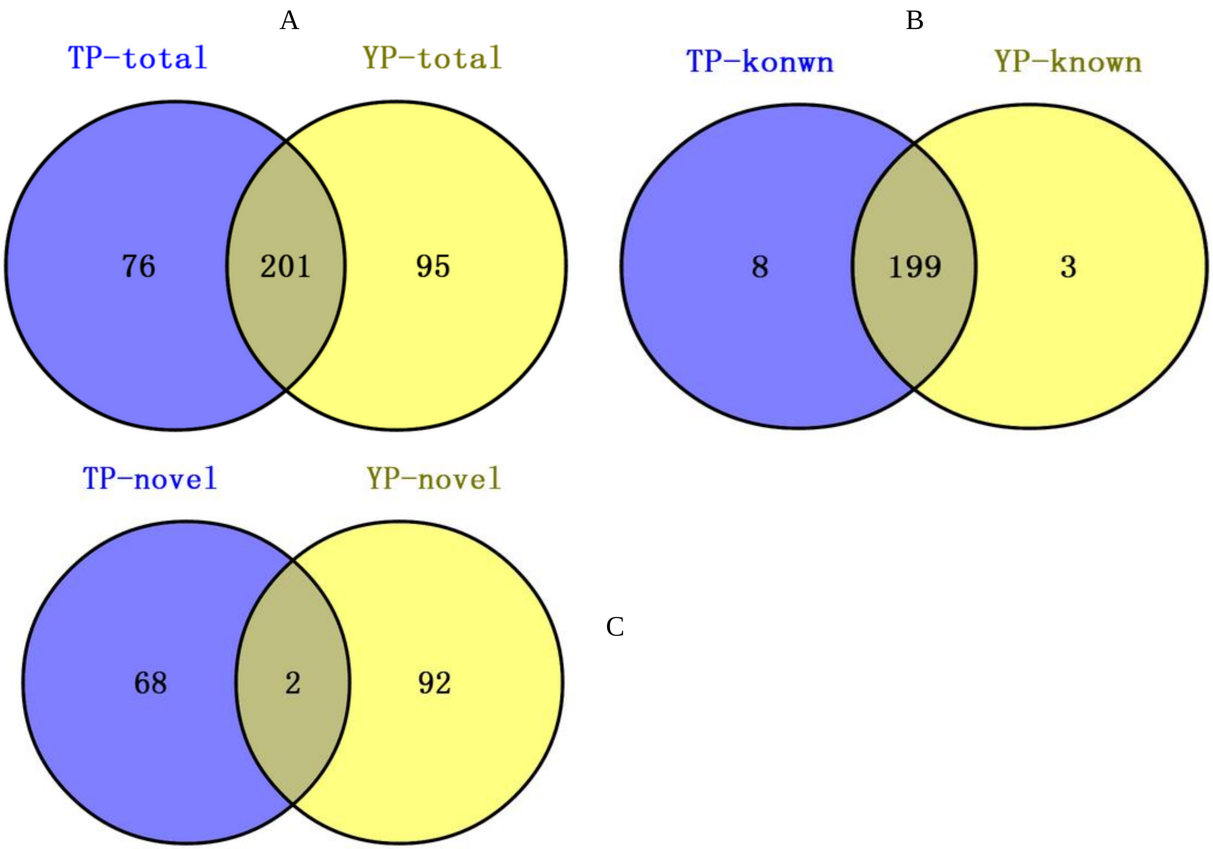

S2 Fig. Venn diagrams demonstrating relationships between miRNAs in Tibetan and Yorkshire pigs.

(A) Venn diagrams for total miRNAs (contained novel miRNAs and known miRNAs). (B) Venn diagrams for known miRNAs. (C) Venn diagrams for novel miRNAs. TP and YP miRNAs marked in blue and yellow cycle, respectively. TP represents Tibetan pigs; YP represents Yorkshire pigs.
