# Supplementary material for: A Comprehensive MicroRNA Expression Profile Related to Hypoxia Adaptation in the Tibetan Pig
Source: PLoS One. 2015 Nov 16;10(11):e0143260. doi: 10.1371/journal.pone.0143260 (PMC4646468; doi:10.1371/journal.pone.0143260)
Supplement: S3 Fig — (PDF) [file pone.0143260.s003.pdf]

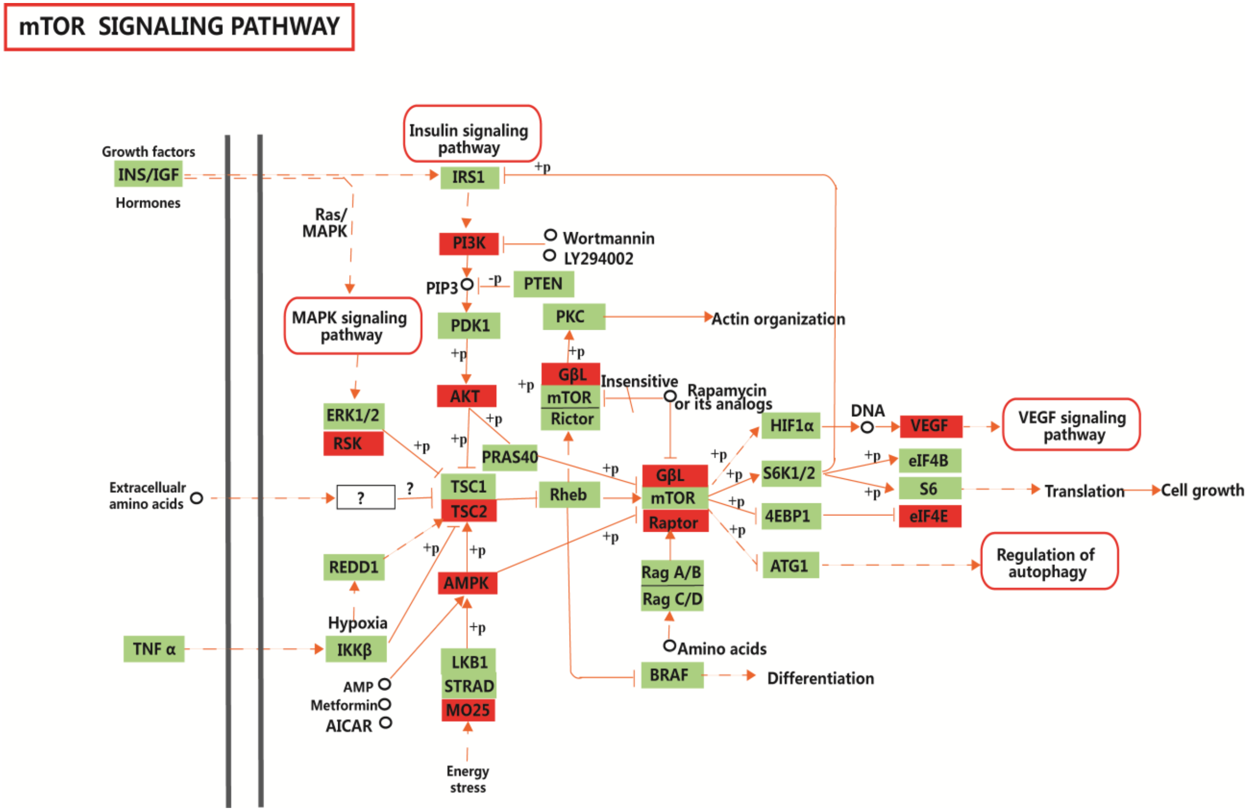

S3 Fig. Mammalian target of the rapamycin (mTOR) signaling pathway enriched by 13 putative target genes of upregulated miRNAs. Red boxes represent the target genes of miRNAs.
