# Supplementary material for: A Comprehensive MicroRNA Expression Profile Related to Hypoxia Adaptation in the Tibetan Pig
Source: PLoS One. 2015 Nov 16;10(11):e0143260. doi: 10.1371/journal.pone.0143260 (PMC4646468; doi:10.1371/journal.pone.0143260)
Supplement: S4 Fig — (PDF) [file pone.0143260.s004.pdf]

A

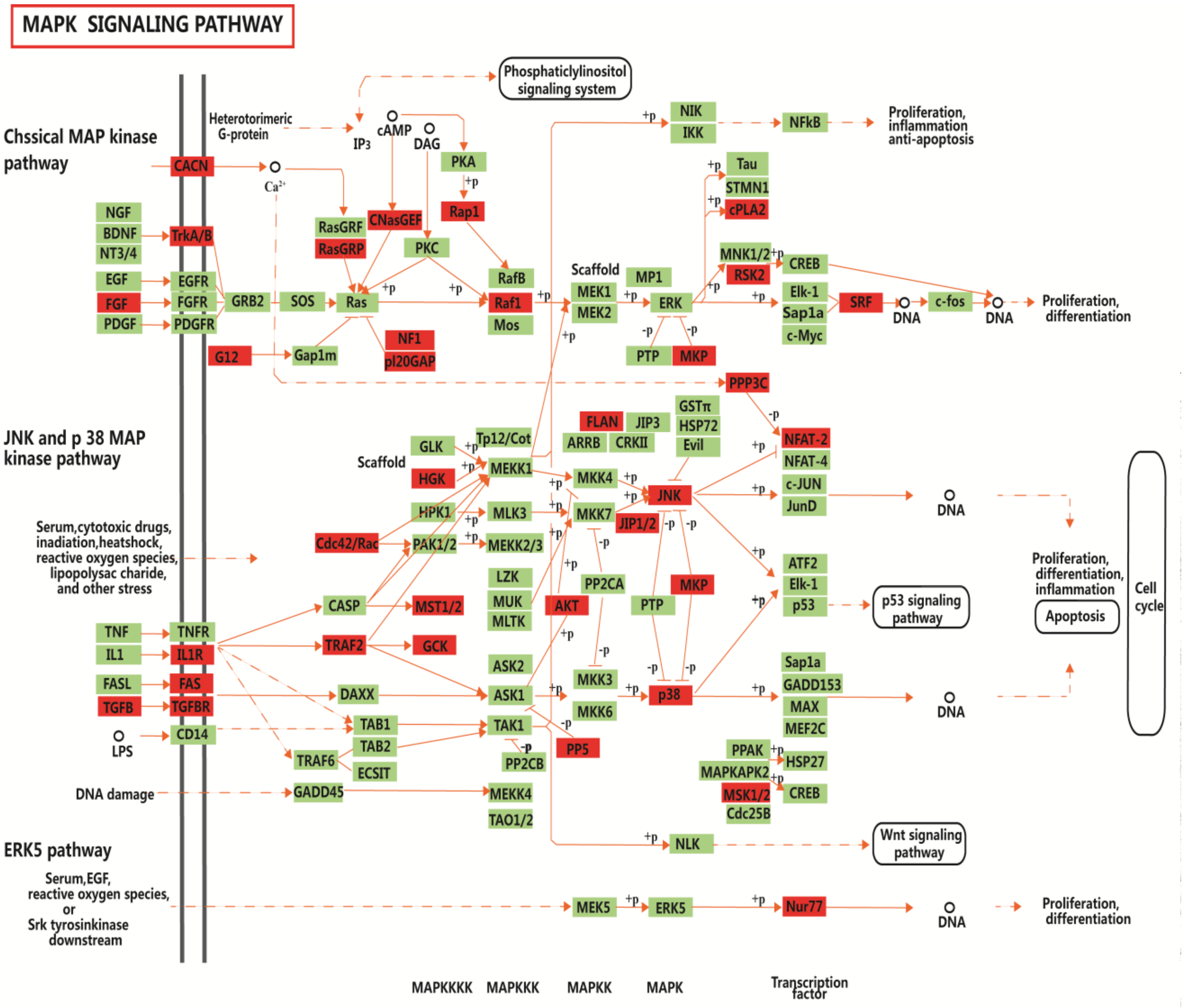
MAPK SIGNALING PATHWAY
Chssical MAP kinase pathway
JNK and p 38 MAP kinase pathway
ERK5 pathway
Heterotorimeric G-protein
Phosphaticlylinositol signaling system
CACN
Ca2+
IP3
cAMP
DAG
PKA
Rap1
NGF
BDNF
NT3/4
TrkA/B
EGF
FGF
PDGF
EGFR
FGFR
PDGFR
GRB2
SOS
Ras
RasGRF
RasGRP
CNasGEF
PKC
RafB
Raf1
Mos
NF1
pl20GAP
G12
Gap1m
Scaffold
MP1
MEK1
MEK2
ERK
PTP
MKP
NIK
IKK
NFkB
Proliferation, inflammation anti-apoptosis
Tau
STMN1
cPLA2
MNK1/2
RSK2
CREB
Elk-1
Sap1a
c-Myc
SRF
c-fos
DNA
Proliferation, differentiation
PPP3C
NFAT-2
NFAT-4
c-JUN
JunD
GSTπ
HSP72
Evil
FLAN
JIP3
ARRB
CRKII
GLK
HGK
Tp12/Cot
MEKK1
HPK1
MLK3
Cdc42/Rac
PAK1/2
MEKK2/3
LZK
MUK
MLTK
MKK4
MKK7
JIP1/2
JNK
PP2CA
AKT
Serum,cytotoxic drugs, inadiation,heatshock, reactive oxygen species, lipopolysac charide, and other stress
CASP
MST1/2
TRAF2
GCK
TNF
IL1
FASL
TGFB
LPS
TNFR
IL1R
FAS
TGFBR
CD14
DAXX
TAB1
TAB2
TRAF6
ECSIT
ASK2
ASK1
TAK1
PP2CB
MKK3
MKK6
p38
PP5
ATF2
Elk-1
p53
p53 signaling pathway
Sap1a
GADD153
MAX
MEF2C
PPAK
MAPKAPK2
MSK1/2
Cdc25B
HSP27
CREB
Proliferation, differentiation, inflammation
Apoptosis
Cell cycle
DNA damage
GADD45
MEKK4
TAO1/2
NLK
Wnt signaling pathway
Serum,EGF, reactive oxygen species, or Srk tyrosinkinase downstream
MEK5
ERK5
Nur77
Proliferation, differentiation
+p
-p
MAPKKKK
MAPKKK
MAPKK
MAPK
Transcription factor

B

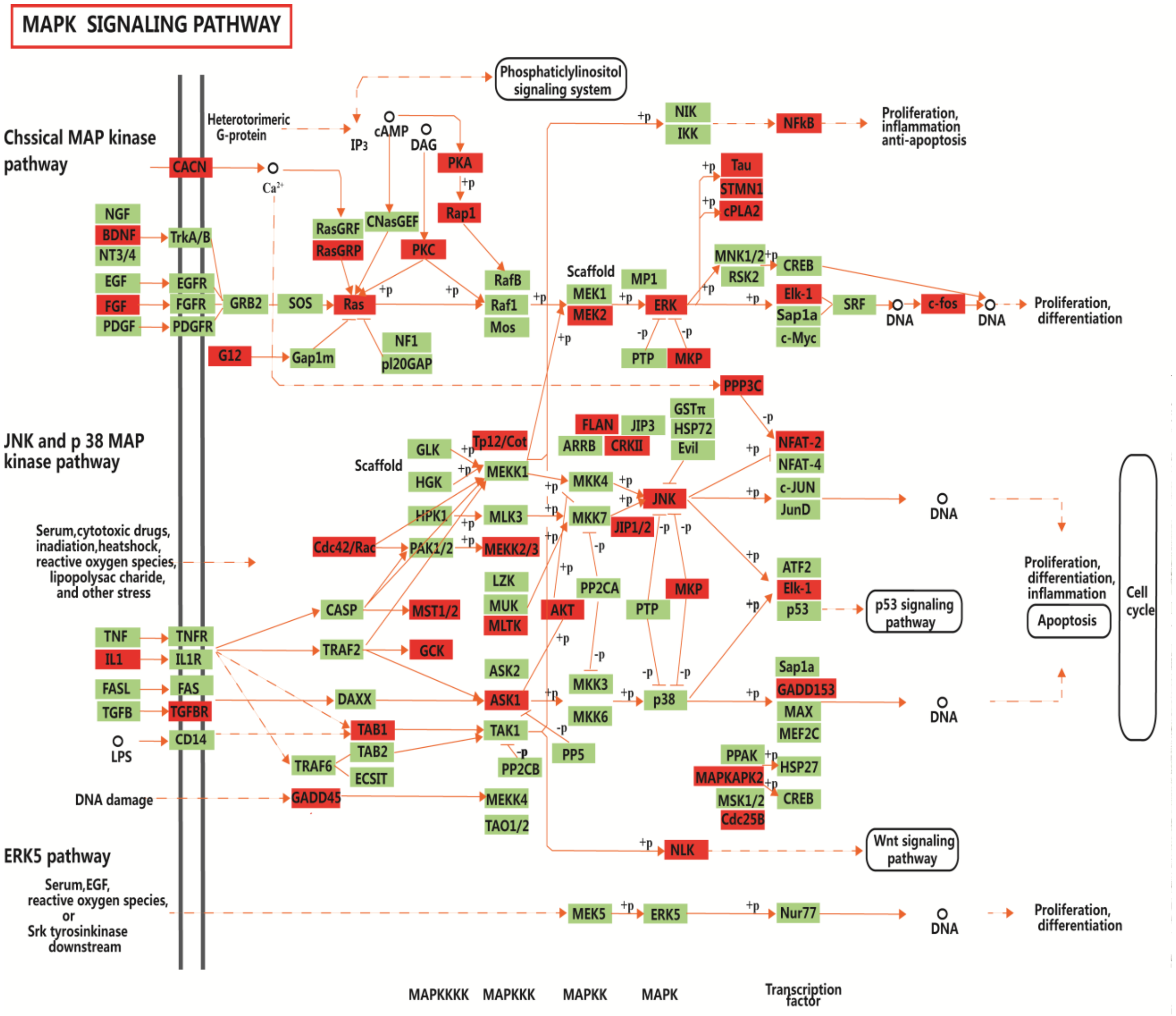
MAPK SIGNALING PATHWAY
Chssical MAP kinase pathway
JNK and p 38 MAP kinase pathway
ERK5 pathway
Heterotorimeric G-protein
Phosphaticlylinositol signaling system
IP3
cAMP
DAG
Ca2+
CACN
PKA
Rap1
NGF
BDNF
NT3/4
TrkA/B
EGF
EGFR
FGF
FGFR
PDGF
PDGFR
GRB2
SOS
RasGRF
RasGRP
CNasGEF
PKC
Ras
G12
Gap1m
NF1
pl20GAP
RafB
Raf1
Mos
Scaffold
MEK1
MEK2
MP1
ERK
PTP
MKP
NIK
IKK
NFkB
Proliferation, inflammation anti-apoptosis
Tau
STMN1
cPLA2
MNK1/2
RSK2
CREB
Elk-1
Sap1a
c-Myc
SRF
c-fos
DNA
Proliferation, differentiation
PPP3C
NFAT-2
NFAT-4
c-JUN
JunD
GSTπ
HSP72
Evil
FLAN
JIP3
ARRB
CRKII
GLK
HGK
Tp12/Cot
MEKK1
HPK1
MLK3
Cdc42/Rac
PAK1/2
MEKK2/3
MKK4
MKK7
JNK
JIP1/2
Serum,cytotoxic drugs, inadiation,heatshock, reactive oxygen species, lipopolysac charide, and other stress
LZK
MUK
MLTK
AKT
PP2CA
MKP
PTP
CASP
MST1/2
TRAF2
GCK
ASK2
ASK1
MKK3
MKK6
p38
TNF
TNFR
IL1
IL1R
FASL
FAS
TGFB
TGFBR
LPS
CD14
DAXX
TAB1
TAB2
TAK1
TRAF6
ECSIT
PP2CB
PP5
ATF2
Elk-1
p53
p53 signaling pathway
Proliferation, differentiation, inflammation
Apoptosis
Cell cycle
Sap1a
GADD153
MAX
MEF2C
PPAK
MAPKAPK2
MSK1/2
Cdc25B
HSP27
CREB
DNA damage
GADD45
MEKK4
TAO1/2
NLK
Wnt signaling pathway
Serum,EGF, reactive oxygen species, or Srk tyrosinkinase downstream
MEK5
ERK5
Nur77
+p
-p
MAPKKKK
MAPKKK
MAPKK
MAPK
Transcription factor

S4 Fig. Mitogen-activated protein kinase (MAPK) signaling pathway enriched by 47 putative target genes of upregulated miRNAs (A) and 53 putative target genes of downregulated miRNAs (B). Red boxes represent the target genes of miRNAs.
