# Supplementary material for: A Comprehensive MicroRNA Expression Profile Related to Hypoxia Adaptation in the Tibetan Pig
Source: PLoS One. 2015 Nov 16;10(11):e0143260. doi: 10.1371/journal.pone.0143260 (PMC4646468; doi:10.1371/journal.pone.0143260)
Supplement: S5 Fig — (PDF) [file pone.0143260.s005.pdf]

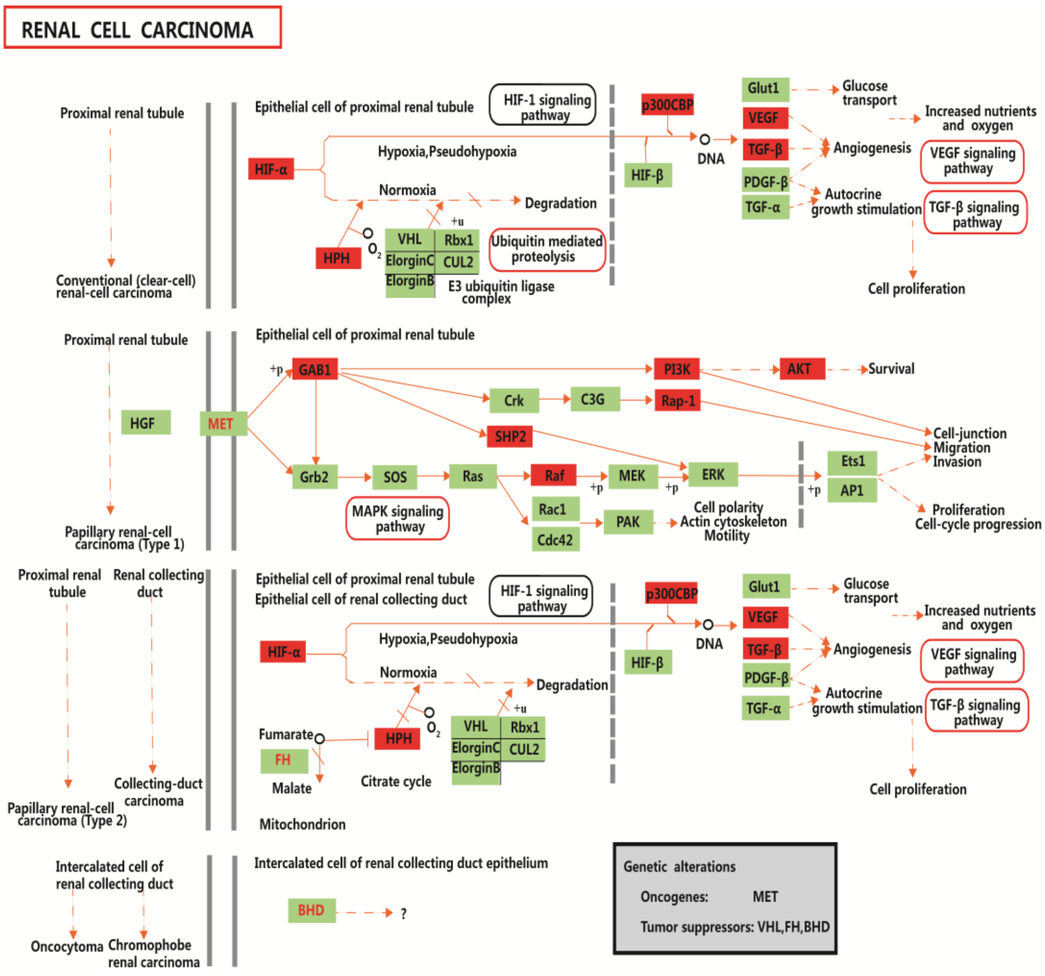

S5 Fig. Renal cell carcinoma pathway enriched by 15 putative target genes of upregulated miRNAs. Red boxes represent the target genes of miRNAs.
